# *N*-[4-Chloro-2-(2-chlorobenzoyl)phenyl]acetamide

**DOI:** 10.1107/S1600536810018696

**Published:** 2010-05-22

**Authors:** F. Nawaz Khan, S. Mohana Roopan, N. Malathi, Venkatesha R. Hathwar, Mehmet Akkurt

**Affiliations:** a Organic and Medicinal Chemistry Research Laboratory, Organic Chemistry Division, School of Advanced Sciences, VIT University, Vellore 632 014, Tamil Nadu, India; b Solid State and Structural Chemistry Unit, Indian Institute of Science, Bangalore 560 012, Karnataka, India; c Department of Physics, Faculty of Arts and Sciences, Erciyes University, 38039 Kayseri, Turkey

## Abstract

In the title compound, C_15_H_11_Cl_2_NO_2_, the dihedral angle between the two benzene rings is 74.83 (5)°. The N-bound and terminal benzene rings are inclined at dihedral angles of 4.09 (10) and 78.38 (9)°, respectively, to the mean plane through the acetamide group. Intra­molecular C—H⋯O and N—H⋯O hydrogen bonds both generate *S*(6) rings.

## Related literature

For the acetyl­ation reaction, see: Greene *et al.* (1999 ▶); Gupta *et al.* (2008 ▶). For solvent-free synthesis, see: Roopan *et al.* (2008 ▶, 2009 ▶). For reactions of acetic anhydride and acetyl chloride, see: Orita *et al.* (2000 ▶); Procopiou *et al.* (1998 ▶). For hydrogen-bond motifs, see: Bernstein *et al.* (1995 ▶).
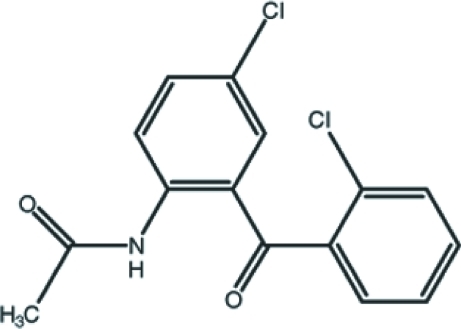


## Experimental

### 

#### Crystal data

C_15_H_11_Cl_2_NO_2_
*M* *_r_* = 308.15
Monoclinic, 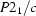

*a* = 11.1371 (11) Å
*b* = 5.0661 (6) Å
*c* = 25.594 (3) Å
β = 100.672 (9)°
*V* = 1419.1 (3) Å^3^
*Z* = 4
Mo *K*α radiation
μ = 0.46 mm^−1^
*T* = 293 K
0.28 × 0.24 × 0.18 mm

#### Data collection

Oxford Xcalibur Eos (Nova) CCD detector diffractometer
14628 measured reflections
2633 independent reflections
1537 reflections with *I* > 2σ(*I*)
*R*_int_ = 0.080

#### Refinement

*R*[*F* ^2^ > 2σ(*F* ^2^)] = 0.048
*wR*(*F* ^2^) = 0.107
*S* = 0.98
2633 reflections
182 parameters
H-atom parameters constrained
Δρ_max_ = 0.18 e Å^−3^
Δρ_min_ = −0.21 e Å^−3^

### 

Data collection: *CrysAlis PRO CCD* (Oxford Diffraction, 2009 ▶); cell refinement: *CrysAlis PRO CCD*; data reduction: *CrysAlis PRO RED* (Oxford Diffraction, 2009 ▶); program(s) used to solve structure: *SHELXS97* (Sheldrick, 2008 ▶); program(s) used to refine structure: *SHELXL97* (Sheldrick, 2008 ▶); molecular graphics: *ORTEP-3 for Windows* (Farrugia, 1997 ▶); software used to prepare material for publication: *WinGX* (Farrugia, 1999 ▶) and *PLATON* (Spek, 2009 ▶).

## Supplementary Material

Crystal structure: contains datablocks global, I. DOI: 10.1107/S1600536810018696/tk2672sup1.cif

Structure factors: contains datablocks I. DOI: 10.1107/S1600536810018696/tk2672Isup2.hkl

Additional supplementary materials:  crystallographic information; 3D view; checkCIF report

## Figures and Tables

**Table 1:** Hydrogen-bond geometry (Å, °)

*D*—H⋯*A*	*D*—H	H⋯*A*	*D*⋯*A*	*D*—H⋯*A*
N1—H1⋯O1	0.86	1.96	2.660 (3)	138
C5—H5⋯O2	0.93	2.22	2.839 (4)	124

**Fig. 1.**
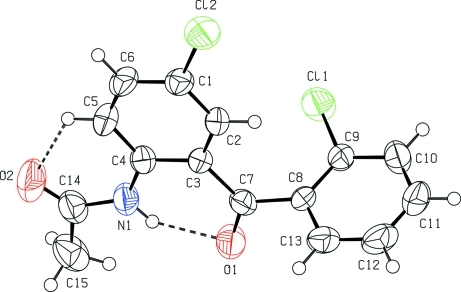
The molecular structure of (I) with the atom numbering scheme. Displacement ellipsoids for non-H atoms are drawn at the 50% probability level.

## supplementary crystallographic information

### Comment

The acetylation of phenol, alcohol and amine are important chemical reactions in organic synthesis (Greene *et al.*, 1999, Gupta *et al.*, 2008). Mainly, acylation of amines is used for the protection of an amino functionality in a multi-step synthetic process. Acetic anhydride and acetyl chloride are generally used in the presence of acidic or basic catalysts in an organic medium (Orita *et al.*, 2000; Procopiou *et al.*, 1998). One of the major factors for a green chemical processes are solvent-free reactions. In continuation of our our interest in this area (Roopan *et al.*, 2008, 2009), we herein report the solvent-free acetylation of an amine, leading to the title compound, (I).

Compound (I), Fig. 1, has two chloro-phenyl groups (Cl2/C1–C6 and Cl1/C8–C13) which make a dihedral angle of 74.83 (5)° with each other. The chloro-phenyl groups are inclined at dihedral angles of 4.09 (10) and 78.38 (9) °, respectively, with the mean plane through the acetamide group (N1/O2/C14/C15). The torsion angles O1—C7—C8—C9, O1—C7—C8—C13, C2—C3—C7—O1 and C4—C3—C7—O1 are 109.0 (3), -68.5 (4), 172.8 (3) and -5.8 (4)°, respectively.

Two intramolecular, i.e. N1—H1···O1 and C5—H5···O2, hydrogen bonds form six-membered rings, producing *S*(6) ring motifs (Table 1, Fig. 1, Bernstein *et al.*, 1995). In the crystal structure, there are no classical intermolecular hydrogen bonds.

### Experimental

2-Amino-5-chloro-phenyl(2-chloro-phenyl)methanone (1 mmol) was stirred with acetyl-chloride (1 mmol) at room temperature for 1 h. The reaction was monitored by TLC. After the completion of the reaction, the contents were cooled and poured onto cold water with stirring. The solid which separated was separated by filtration and dried in air. The dried compound was dissolved in dichloromethane and subjected to slow evaporation to yield single crystals.

### Refinement

All the H atoms were discernible in the difference Fourier maps. However, H atoms were located geometrically with N—H = 0.86 and C—H = 0.93-0.96 Å and refined in the riding model approximation, with *U*_iso_(H) = 1.2 or 1.5*U*_eq_(C, N).

### Crystal data

**Table:**

C_15_H_11_Cl_2_NO_2_	*F*(000) = 632
*M**_r_* = 308.15	*D*_x_ = 1.442 Mg m^−^^3^
Monoclinic, *P*2_1_/*c*	Mo *K*α radiation, λ = 0.71073 Å
Hall symbol: -P 2ybc	Cell parameters from 1298 reflections
*a* = 11.1371 (11) Å	θ = 2.0–20.9°
*b* = 5.0661 (6) Å	µ = 0.46 mm^−^^1^
*c* = 25.594 (3) Å	*T* = 293 K
β = 100.672 (9)°	Block, colourless
*V* = 1419.1 (3) Å^3^	0.28 × 0.24 × 0.18 mm
*Z* = 4	

### Data collection

**Table:**

Oxford Xcalibur Eos (Nova) CCD detector diffractometer	1537 reflections with *I* > 2σ(*I*)
Radiation source: Enhance (Mo) X-ray Source	*R*_int_ = 0.080
graphite	θ_max_ = 25.5°, θ_min_ = 2.7°
ω scans	*h* = −13→13
14628 measured reflections	*k* = −6→6
2633 independent reflections	*l* = −30→30

### Refinement

**Table:**

Refinement on *F*^2^	Primary atom site location: structure-invariant direct methods
Least-squares matrix: full	Secondary atom site location: difference Fourier map
*R*[*F*^2^ > 2σ(*F*^2^)] = 0.048	Hydrogen site location: inferred from neighbouring sites
*wR*(*F*^2^) = 0.107	H-atom parameters constrained
*S* = 0.98	*w* = 1/[σ^2^(*F*_o_^2^) + (0.0469*P*)^2^] where *P* = (*F*_o_^2^ + 2*F*_c_^2^)/3
2633 reflections	(Δ/σ)_max_ < 0.001
182 parameters	Δρ_max_ = 0.18 e Å^−^^3^
0 restraints	Δρ_min_ = −0.21 e Å^−^^3^

### Special details

**Table:**

Geometry. Bond distances, angles etc. have been calculated using the rounded fractional coordinates. All su's are estimated from the variances of the (full) variance-covariance matrix. The cell esds are taken into account in the estimation of distances, angles and torsion angles
Refinement. Refinement on F^2^ for ALL reflections except those flagged by the user for potential systematic errors. Weighted R-factors wR and all goodnesses of fit S are based on F^2^, conventional R-factors R are based on F, with F set to zero for negative F^2^. The observed criterion of F^2^ > 2sigma(F^2^) is used only for calculating -R-factor-obs etc. and is not relevant to the choice of reflections for refinement. R-factors based on F^2^ are statistically about twice as large as those based on F, and R-factors based on ALL data will be even larger.

### Fractional atomic coordinates and isotropic or equivalent isotropic displacement parameters (Å^2^)

**Table:**

	*x*	*y*	*z*	*U*_iso_*/*U*_eq_	
Cl1	1.01228 (8)	0.21348 (16)	0.05190 (3)	0.0676 (3)	
Cl2	0.68968 (8)	0.97146 (17)	0.00452 (3)	0.0706 (3)	
O1	0.94298 (18)	0.2513 (5)	0.19297 (8)	0.0777 (9)	
O2	0.5030 (2)	0.0779 (5)	0.17216 (10)	0.0936 (11)	
N1	0.7034 (2)	0.1634 (5)	0.17250 (9)	0.0533 (9)	
C1	0.6927 (2)	0.7372 (6)	0.05413 (10)	0.0477 (10)	
C2	0.8029 (2)	0.6603 (5)	0.08384 (10)	0.0429 (9)	
C3	0.8082 (2)	0.4689 (5)	0.12330 (10)	0.0396 (9)	
C4	0.6978 (2)	0.3551 (5)	0.13289 (10)	0.0431 (10)	
C5	0.5876 (3)	0.4396 (6)	0.10239 (12)	0.0580 (11)	
C6	0.5855 (3)	0.6267 (6)	0.06384 (12)	0.0566 (11)	
C7	0.9300 (2)	0.3982 (6)	0.15419 (11)	0.0459 (10)	
C8	1.0428 (2)	0.5175 (5)	0.13994 (10)	0.0399 (9)	
C9	1.0892 (2)	0.4451 (5)	0.09588 (10)	0.0435 (10)	
C10	1.1978 (3)	0.5487 (6)	0.08559 (12)	0.0565 (11)	
C11	1.2597 (3)	0.7314 (7)	0.11949 (14)	0.0661 (13)	
C12	1.2151 (3)	0.8101 (7)	0.16279 (13)	0.0686 (12)	
C13	1.1075 (3)	0.7045 (6)	0.17374 (11)	0.0586 (11)	
C14	0.6092 (3)	0.0330 (6)	0.18928 (13)	0.0607 (12)	
C15	0.6499 (3)	−0.1678 (7)	0.23219 (13)	0.0775 (16)	
H1	0.77560	0.12130	0.18860	0.0640*	
H2	0.87480	0.73680	0.07750	0.0510*	
H5	0.51450	0.36760	0.10840	0.0700*	
H6	0.51110	0.68050	0.04390	0.0680*	
H10	1.22820	0.49410	0.05590	0.0680*	
H11	1.33250	0.80190	0.11290	0.0790*	
H12	1.25710	0.93660	0.18540	0.0820*	
H13	1.07860	0.75900	0.20380	0.0700*	
H15A	0.63180	−0.10450	0.26520	0.1160*	
H15B	0.73630	−0.19610	0.23590	0.1160*	
H15C	0.60750	−0.33100	0.22290	0.1160*	

### Atomic displacement parameters (Å^2^)

**Table:**

	*U*^11^	*U*^22^	*U*^33^	*U*^12^	*U*^13^	*U*^23^
Cl1	0.0736 (6)	0.0686 (6)	0.0632 (5)	−0.0187 (5)	0.0197 (4)	−0.0213 (4)
Cl2	0.0681 (6)	0.0796 (6)	0.0622 (5)	0.0248 (5)	0.0069 (4)	0.0221 (5)
O1	0.0495 (13)	0.117 (2)	0.0670 (14)	0.0078 (13)	0.0115 (11)	0.0456 (14)
O2	0.0531 (15)	0.121 (2)	0.109 (2)	−0.0246 (16)	0.0209 (14)	0.0138 (17)
N1	0.0417 (14)	0.0620 (17)	0.0590 (16)	−0.0031 (13)	0.0164 (12)	0.0079 (14)
C1	0.0450 (18)	0.054 (2)	0.0428 (16)	0.0096 (15)	0.0050 (14)	−0.0018 (14)
C2	0.0343 (15)	0.0506 (18)	0.0446 (16)	0.0030 (13)	0.0098 (13)	−0.0003 (15)
C3	0.0334 (15)	0.0486 (18)	0.0367 (15)	0.0029 (13)	0.0061 (12)	0.0003 (14)
C4	0.0394 (16)	0.0466 (18)	0.0446 (16)	0.0048 (14)	0.0110 (13)	−0.0019 (15)
C5	0.0325 (16)	0.077 (2)	0.064 (2)	0.0005 (16)	0.0074 (15)	−0.0017 (19)
C6	0.0357 (17)	0.075 (2)	0.0552 (19)	0.0166 (16)	−0.0017 (14)	−0.0033 (18)
C7	0.0460 (17)	0.056 (2)	0.0368 (16)	0.0067 (15)	0.0108 (13)	0.0062 (15)
C8	0.0304 (14)	0.0515 (19)	0.0357 (15)	0.0058 (14)	0.0004 (12)	0.0049 (14)
C9	0.0392 (16)	0.0481 (18)	0.0427 (16)	−0.0041 (14)	0.0066 (13)	−0.0055 (14)
C10	0.0502 (19)	0.064 (2)	0.060 (2)	−0.0023 (17)	0.0223 (16)	−0.0031 (17)
C11	0.0431 (18)	0.081 (3)	0.072 (2)	−0.0194 (19)	0.0052 (17)	0.001 (2)
C12	0.066 (2)	0.071 (2)	0.061 (2)	−0.022 (2)	−0.0085 (18)	−0.0118 (19)
C13	0.060 (2)	0.071 (2)	0.0425 (17)	−0.0004 (18)	0.0032 (15)	−0.0111 (17)
C14	0.062 (2)	0.066 (2)	0.059 (2)	−0.0170 (19)	0.0242 (18)	−0.0106 (18)
C15	0.098 (3)	0.071 (3)	0.071 (2)	−0.026 (2)	0.035 (2)	0.003 (2)

### Geometric parameters (Å, °)

**Table:**

Cl1—C9	1.738 (3)	C8—C9	1.374 (3)
Cl2—C1	1.734 (3)	C9—C10	1.388 (4)
O1—C7	1.228 (4)	C10—C11	1.365 (5)
O2—C14	1.205 (4)	C11—C12	1.356 (5)
N1—C4	1.397 (3)	C12—C13	1.388 (5)
N1—C14	1.374 (4)	C14—C15	1.504 (5)
N1—H1	0.8600	C2—H2	0.9300
C1—C6	1.382 (4)	C5—H5	0.9300
C1—C2	1.375 (3)	C6—H6	0.9300
C2—C3	1.393 (4)	C10—H10	0.9300
C3—C4	1.420 (3)	C11—H11	0.9300
C3—C7	1.482 (3)	C12—H12	0.9300
C4—C5	1.394 (4)	C13—H13	0.9300
C5—C6	1.365 (4)	C15—H15A	0.9600
C7—C8	1.499 (3)	C15—H15B	0.9600
C8—C13	1.391 (4)	C15—H15C	0.9600
			
Cl1···C2	3.455 (3)	C13···O1^x^	3.407 (4)
Cl1···C3	3.423 (3)	C7···H1	2.5000
Cl1···Cl1^i^	3.3974 (12)	C8···H2	2.4900
Cl2···C11^ii^	3.650 (4)	C9···H2	2.7700
Cl1···H2^iii^	3.0000	C12···H15A^xi^	3.0900
Cl2···H6^iv^	2.9400	C14···H5	2.7300
Cl2···H10^v^	3.0500	C15···H12^xii^	2.9500
O1···N1	2.660 (3)	H1···O1	1.9600
O1···C13^iii^	3.407 (4)	H1···C7	2.5000
O2···C5	2.839 (4)	H1···H15B	2.1100
O2···C11^vi^	3.300 (4)	H2···Cl1^x^	3.0000
O1···H13^vii^	2.7000	H2···C8	2.4900
O1···H1	1.9600	H2···C9	2.7700
O1···H13^iii^	2.9000	H5···O2	2.2200
O2···H5	2.2200	H5···C14	2.7300
O2···H11^vi^	2.6100	H6···Cl2^iv^	2.9400
O2···H12^vi^	2.9100	H10···Cl2^v^	3.0500
O2···H15A^viii^	2.8800	H11···O2^ix^	2.6100
N1···O1	2.660 (3)	H12···O2^ix^	2.9100
C2···Cl1	3.455 (3)	H12···C15^xiii^	2.9500
C2···C9	3.330 (3)	H13···O1^x^	2.9000
C3···Cl1	3.423 (3)	H13···O1^xi^	2.7000
C5···O2	2.839 (4)	H15A···O2^xiv^	2.8800
C9···C2	3.330 (3)	H15A···C12^vii^	3.0900
C11···O2^ix^	3.300 (4)	H15B···H1	2.1100
C11···Cl2^ii^	3.650 (4)		
			
C4—N1—C14	128.8 (2)	C11—C12—C13	120.8 (3)
C14—N1—H1	116.00	C8—C13—C12	120.3 (3)
C4—N1—H1	116.00	N1—C14—C15	114.2 (3)
Cl2—C1—C6	120.6 (2)	O2—C14—N1	123.4 (3)
C2—C1—C6	119.9 (3)	O2—C14—C15	122.4 (3)
Cl2—C1—C2	119.55 (19)	C1—C2—H2	120.00
C1—C2—C3	120.8 (2)	C3—C2—H2	120.00
C2—C3—C7	117.8 (2)	C4—C5—H5	120.00
C2—C3—C4	119.1 (2)	C6—C5—H5	120.00
C4—C3—C7	123.1 (2)	C1—C6—H6	120.00
N1—C4—C5	122.4 (2)	C5—C6—H6	120.00
C3—C4—C5	118.6 (2)	C9—C10—H10	120.00
N1—C4—C3	119.0 (2)	C11—C10—H10	120.00
C4—C5—C6	120.9 (3)	C10—C11—H11	120.00
C1—C6—C5	120.8 (3)	C12—C11—H11	120.00
O1—C7—C3	122.5 (2)	C11—C12—H12	120.00
C3—C7—C8	119.9 (2)	C13—C12—H12	120.00
O1—C7—C8	117.6 (2)	C8—C13—H13	120.00
C7—C8—C13	119.0 (2)	C12—C13—H13	120.00
C9—C8—C13	117.5 (2)	C14—C15—H15A	109.00
C7—C8—C9	123.5 (2)	C14—C15—H15B	110.00
C8—C9—C10	121.9 (2)	C14—C15—H15C	109.00
Cl1—C9—C8	119.76 (18)	H15A—C15—H15B	109.00
Cl1—C9—C10	118.4 (2)	H15A—C15—H15C	109.00
C9—C10—C11	119.4 (3)	H15B—C15—H15C	110.00
C10—C11—C12	120.0 (3)		
			
C14—N1—C4—C3	178.3 (3)	N1—C4—C5—C6	−179.7 (3)
C14—N1—C4—C5	−1.6 (4)	C3—C4—C5—C6	0.4 (4)
C4—N1—C14—O2	−3.1 (5)	C4—C5—C6—C1	−0.1 (5)
C4—N1—C14—C15	178.4 (3)	O1—C7—C8—C9	109.0 (3)
Cl2—C1—C2—C3	−178.8 (2)	O1—C7—C8—C13	−68.5 (4)
C6—C1—C2—C3	0.9 (4)	C3—C7—C8—C9	−73.8 (4)
Cl2—C1—C6—C5	179.1 (2)	C3—C7—C8—C13	108.7 (3)
C2—C1—C6—C5	−0.6 (4)	C7—C8—C9—Cl1	2.8 (4)
C1—C2—C3—C4	−0.6 (4)	C7—C8—C9—C10	−176.2 (3)
C1—C2—C3—C7	−179.3 (2)	C13—C8—C9—Cl1	−179.7 (2)
C2—C3—C4—N1	−180.0 (2)	C13—C8—C9—C10	1.4 (4)
C2—C3—C4—C5	−0.1 (4)	C7—C8—C13—C12	177.2 (3)
C7—C3—C4—N1	−1.3 (4)	C9—C8—C13—C12	−0.4 (4)
C7—C3—C4—C5	178.5 (3)	Cl1—C9—C10—C11	179.8 (2)
C2—C3—C7—O1	172.8 (3)	C8—C9—C10—C11	−1.2 (4)
C2—C3—C7—C8	−4.3 (4)	C9—C10—C11—C12	0.0 (5)
C4—C3—C7—O1	−5.8 (4)	C10—C11—C12—C13	0.9 (5)
C4—C3—C7—C8	177.1 (2)	C11—C12—C13—C8	−0.7 (5)

Symmetry codes: (i) −*x*+2, −*y*, −*z*; (ii) −*x*+2, −*y*+2, −*z*; (iii) *x*, *y*−1, *z*; (iv) −*x*+1, −*y*+2, −*z*; (v) −*x*+2, −*y*+1, −*z*; (vi) *x*−1, *y*−1, *z*; (vii) −*x*+2, *y*−1/2, −*z*+1/2; (viii) −*x*+1, *y*+1/2, −*z*+1/2; (ix) *x*+1, *y*+1, *z*; (x) *x*, *y*+1, *z*; (xi) −*x*+2, *y*+1/2, −*z*+1/2; (xii) −*x*+2, *y*−3/2, −*z*+1/2; (xiii) −*x*+2, *y*+3/2, −*z*+1/2; (xiv) −*x*+1, *y*−1/2, −*z*+1/2.

### Hydrogen-bond geometry (Å, °)

**Table:**

*D*—H···*A*	*D*—H	H···*A*	*D*···*A*	*D*—H···*A*
N1—H1···O1	0.86	1.96	2.660 (3)	138
C5—H5···O2	0.93	2.22	2.839 (4)	124
